# National Cemetery

**Published:** 1830-10-01

**Authors:** 


### National Cemetery.

At the present time, when all London is agog to be buried in the prettiest possible way, and the periodical prints seem likely to become little else than the epitaphs of their editors, the opinions of the learned Sir Thomas Browne are not mal-à-propos. He observes that in olden times, "when the funeral pyre was out, and the last valediction over, men took a lasting adieu of their interred friends, little expecting the curiosity of future ages should comment upon their ashes; and having no old experience of the duration of their reliques, held no opinion of such after consideration. But who knows the fate of his bones, or how often he is to be buried?"

He thinks that the practice of burning and burying the body were equally ancient. According to some tradition, Adam was buried near Damascus, or Mount Calvary; and Abraham and the patriachs were also buried. Hector was burned before the gates of Troy. Among the Romans, Manlius, the consul, burnt the body of his son; but Numa, by a special clause in his will, was not burnt, but buried; and Remus was also solemnly buried. The two ceremonies seem, therefore, to have been coeval and indifferent. The origin of *cremation*, or burning, he thinks, may be attributed to the opinions of those ancient philosophers who conceived that fire was the master principle in the composition of our bodies; and, therefore, funeral piles were heaped up, in order to waft them more speedily to their native element. But the Indian Brahmins, he is rather disposed to think, "are too great friends unto fire, for they imagine it the noblest way to end their days in fire, and therefore burn themselves alive." He mentions the different modes of burying as practised by various nations, and remarks that the rites of sepulture do not seem to be confined to man, for there would appear to be some approach to this practice among elephants, cranes, ants, and bees; "the latter civil society," says Browne, "at least carry out their dead, and hath exequies, if not interments."

Sir Thomas, on the whole, concludes in favour of *cremation*, or burning; for says he, "to be knaved out of our graves, to have our skulls made drinking bowls, and our bones turned into pipes, to delight and sport our enemies, are tragical abominations, escaped in burning burials." We know not what our contemporary, the Gazette, will say to this, when it sadly complains of the present effluvia from the city churchyards being quite insupportable to its olfactories. The sulphuretted hydrogen disengaged from a London *cremation*, as Sir Thomas has it, would induce lypothymia, or even turn the green of its cover black. We would strongly recommend the directors of the London Pére-la-Chaise, to keep a pyre in some part of their grounds for those who have a yearning after the customs of antiquity. Librarians, school-masters, and those sunk deep in the *dead* languages, might still be inurned, and like Martin Scriblerus imagine themselves the veritable classical Amphitryon.

